# Acute proximal left anterior descending thrombosis manifested by persistent hiccups: A case report

**DOI:** 10.1097/MD.0000000000018096

**Published:** 2019-11-27

**Authors:** Hanxiang Gao, Bo Zhang, Li Song, Suyu Yao, Zheng Zhang, Ming Bai

**Affiliations:** Department of Cardiology, First Hospital of Lanzhou University, Lanzhou 730000, Gansu, China.

**Keywords:** case report, coronary artery disease, hiccups, thrombosis

## Abstract

**Introduction:**

Hiccup is usually a benign and self-limited phenomenon. Therefore, if hiccups do not resolve by themselves and even last for a long time, it may be the marker of serious medical conditions.

**Patient concerns:**

We encountered a case presenting with recurrent abdominal discomfort. Diffuse ST-segment elevation in V2-V6 and elevated Troponin I was identified. He had complained about constipation and incomplete intestinal obstruction was ever suspected. Four days later, he exhibited persistent hiccups.

**Diagnosis:**

He was diagnosed with acute anterior wall myocardial infarction. And elective coronary angiography showed that proximal left anterior descending (LAD) was occluded by fresh thrombus with TIMI 1 flow.

**Interventions:**

The lesion in proximal LAD was dilated with low pressure. Interestingly, the hiccups reduced. And after stent implantation the hiccup disappeared in 24 hours.

**Outcomes:**

The patient was discharged in good general condition, with maintenance therapy and a follow-up protocol.

**Conclusion:**

Hiccup is only rarely described in the context of acute proximal LAD thrombosis. However, if this special symptom occurs intractably during disease progression, thrombus is revealed to be the probable cause, prompt opening of the criminal vessel should be performed besides strengthening of anticoagulant and antiplatelet.

## 1 Introduction

Hiccup is a spasmodic involuntary contraction of the diaphragm and accessory muscles of inspiration, immediately followed by glottis closure terminating inspiration.^[1]^ It is usually a benign and self-limited phenomenon. Therefore, if hiccups do not resolve by themselves and even last for a long time, it may be the marker of serious medical conditions. Hiccups could be induced by lots of potential reasons. Digestive tract disorders are the most common etiologies of intractable hiccups, and it accounting for 62.5% of the hospitalized patients.^[2]^ In this article, we describe an acute myocardial infarction (AMI) patient with proximal left anterior descending (LAD) thrombosis, presenting with persistent hiccups, which alleviate after effective treatment of the thrombosis.

## 2 Case report

A 46-year-old male presented with recurrent abdominal discomfort for 2 days. He had smoked for 20 years and had prior 2 years history of ischemic stroke with left hemiparesis. Cholecystitis was identified by abdominal ultrasonography. The electrocardiogram (ECG) detected diffuse ST-segment elevation in V2-V6, inverted T-wave in V3R-V5R, and complete right bundle branch block. Both creatine kinase isoenzyme and troponin I was highly elevated. He had been treated with aspirin and antibiotics before transferred to our institute 24 hours later.

At the admission, he still had abdominal sign, nausea, and anorexia. Laboratory results showed mild hypokalemia (3.3 mmol/L). Aminotransferase and bilirubin were consistently high. International normalized ratio (INR) was 1.25. Tumor markers and serology for hepatitis were negative. Hypokalemia was corrected by oral potassium citrate and potassium ranged between 3.65–4.5 mmol/L. Premature ventricular contraction and non-sustained ventricular tachycardia could be observed every day through ECG monitor system. Meanwhile, he was given polyene phosphatidyl choline and glucurolactone for the abnormal liver function. Besides, he was instructed to take daily aspirin 100 mg, clopidogrel 75 mg, statin 40 mg, beta-blockers 5 mg, and proton pump inhibitors 40 mg.

He had complained about constipation twice, both of which were alleviated by glycerine enema injection. Incomplete intestinal obstruction was ever suspected after consulted with gastroenterologist due to appearance of gastrointestinal symptoms. Four days later, he exhibited hiccups, which could be aggravated by drinking. All the treatment including glottis stimulation, breath holding, acupoint massage, and enema were unfortunately ineffective. Gastric motility drugs and digestive enzymes could not resolve the hiccups either.

Six days post admission he collapsed suddenly and the remote ECG monitor system showed a ventricular fibrillation. A sinus rhythm returned after defibrillation. His potassium was 3.3 mmol/L. Intravenous amiodarone and potassium chloride was initiated. Heart rate was improved to around 70–80 beats per minute, potassium was held between 3.78 and 4.03 mmol/L and QT interval was kept less than 0.46 ms. However, ventricular arrhythmia happened for another 5 times in the next 60 hours with 3 times happened in the early morning. And hiccups persisted during this period except within 30 minutes after the use of defibrillator each time.

**Figure 1:**
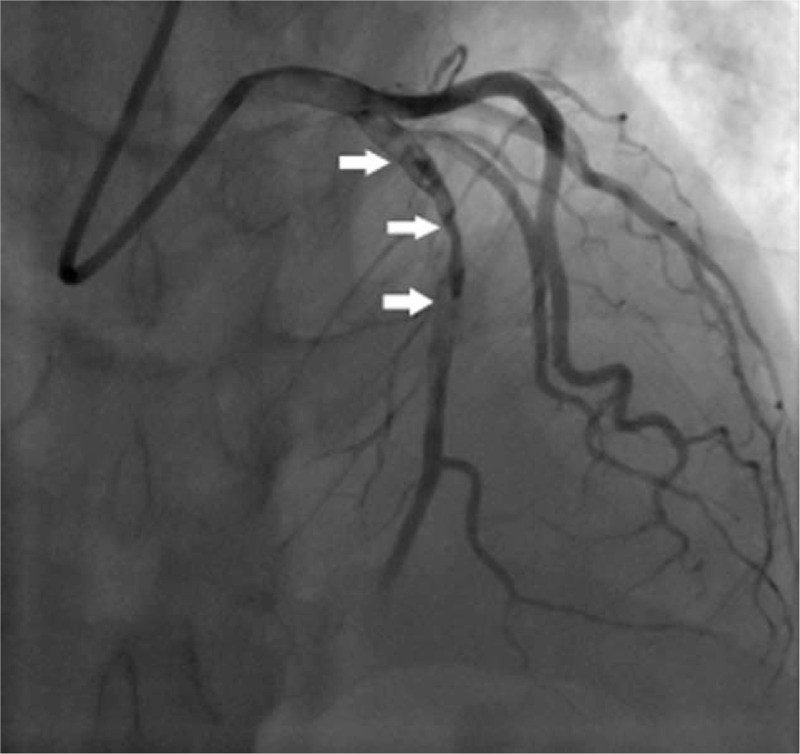
LAD injection shows occlusion of proximal part by fresh thrombus (arrow). LAD = left anterior descending.

**Figure 2:**
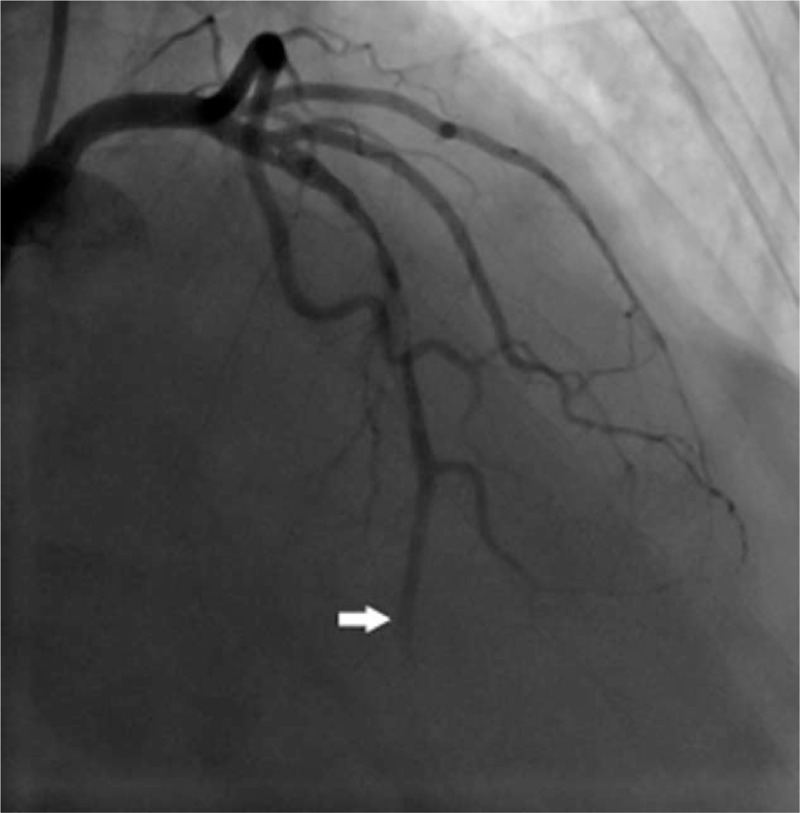
LAD injection after thrombus aspiration shows TIMI I flow. LAD = left anterior descending.

Elective coronary angiography was performed via the trans-radial route 9 days after the admission. It showed that proximal LAD was occluded by fresh thrombus with TIMI 1 flow (Fig. 1). Left main coronary artery, left circumflex artery, and right coronary artery were completely normal. The LAD lesion was crossed using a guide wire and dilated with low pressure. Then TIMI 1 flow was achieved with a thrombus noticed in the proximal LAD, after which aspiration catheter was ran repeatedly but TIMI flow did not improve any more (Fig. 2). The patient was transferred back to cardiac care unit. Following the clinical and angiographic findings, 8000 units low molecular weight heparin, 100 mg of aspirin, and 150 mg of clopidogrel were administered for 5 days. Interestingly, the hiccups reduced.

**Figure 3:**
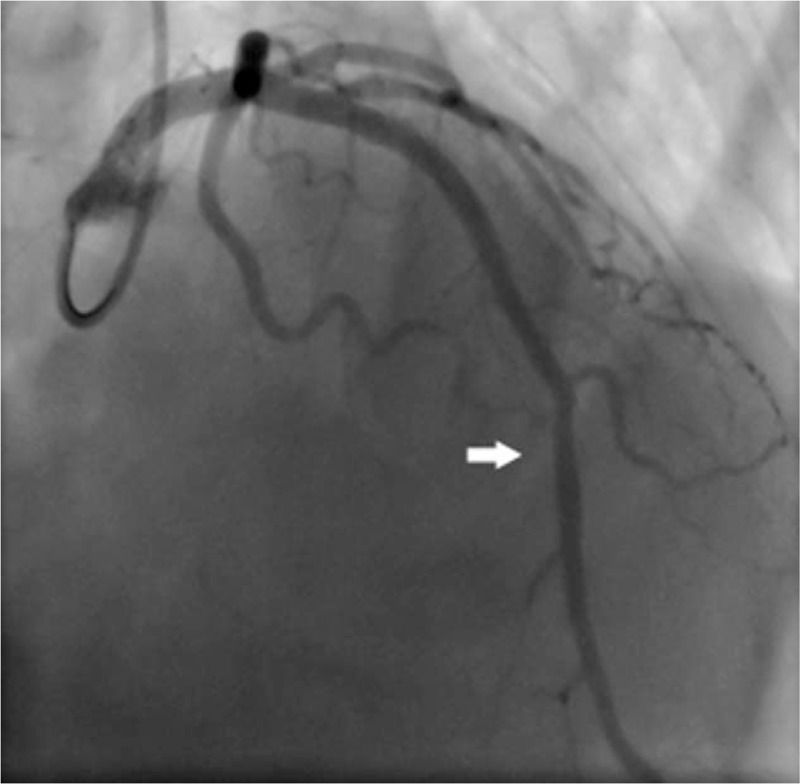
LAD injection post-stenting shows adequate TIMI III flow. The stent is illustrated by the arrow. LAD = left anterior descending.

Another 5 days later, percutaneous coronary intervention was decided. Repeat coronary angiography showed LAD with TIMI 2 flow. After wiring and dilatation of the proximal LAD using a 2.5 mm × ∗20 mm TREK balloon, thrombus aspiration was performed (Export AP 6F aspiration catheter) for 6 times. Several thrombotic materials were noticed in the collection cup, and the TIMI flow improved to grade 3 (Fig. 3). Two stents were placed overlapped in the site. And after subsequent medical therapy the hiccup disappeared in 24 hours. The patient was discharged in good general condition, with maintenance therapy and a follow-up protocol.

## 3 Discussion

Hiccups are a common physiological feature. Therefore, when it becomes persistent even intractable, it is probably induced by potential pathophysiological processes that affect a component of the hiccup reflex mechanism.^[1]^ Hiccups could be induced by lots of potential reasons. Digestive tract disorders are the most common etiologies of intractable hiccups, and it accounting for 62.5% of the hospitalized patients.^[2]^ A case report indicated that non-erosive reflex disease could also manifested by hiccups exclusively.^[1]^ Hiccups are not rare to be detected in people with esophageal cancer. It had been revealed that hiccups occurred in 27% primary esophageal patients and lasted for more than 48 hours.^[3]^ Hiccups had been considered as a subtle sign of gastric volvulus as well.^[4]^ Digestive system disorders are initial symptoms of our patient. He had persistent abdominal discomfort besides nausea and constipation. In this case strict bed rest is another culprit for digestive problems. Therefore, incomplete intestinal obstruction could not be ruled out. And then hiccups occurred 6 days later. It is also unbelievable that ileus was indicated as a cause of hiccup, which was not confirmed for lack of imaging examination. Series of gastrointestinal symptoms appeared in this patient and no response to spasmolysis and adjustment of gastrointestinal motility.

Hiccupping is known to be composed of several neural pathways, which contain central nervous system.^[5]^ Vascular, infectious or structural processes are supposed to suppress the hiccup reflex. There is no doubt that ischemic stroke has the chance to elicit hiccup. Nevertheless, the symptom always resolved after correct diagnosis and treatment. Hiccup was also reported as a subtle sign of neuromyelitis optica besides nausea and vomiting.^[6]^ Brain tumors and cerebellar artery aneurysm were also considered as the obvious cause of hiccup.^[7]^ In the present case, apparent 2-year history of ischemic stroke was extracted, and he did not experience any gastrointestinal symptoms before hospitalization this time. What's more, no evidence of new cerebral infarction was detected while in hospital. Hence the relationship between stroke and hiccups could not be established in our patient.

It is confirmed that myocardial ischemia was indicated as an uncommon cause of hiccup, which was indicated via successful coronary intervention therapy.^[8]^ Joshua Davenport et al even suggested that hiccups could be present as the only symptom of non-ST-segment elevation myocardial infarction.^[9]^ A patient was confirmed to manifest persistent hiccups after cardiorespiratory arrest.^[10]^ This particular patient displayed downward frequency of singultus in response to dilatation of the proximal LAD with low pressure. When combined with enhanced anticoagulant therapy, symptoms were alleviated obviously in the next few days. Finally our patient was successfully treated by thrombus aspiration and stent implantation. In the present study, eventual symptom resolution after improvement of coronary blood flow could certify the association between hiccups and myocardial ischemia.

LAD plays a major role in myocardial blood supply. The occlusion of LAD can lead to massive infarctions including left anterior ventricular wall, cardiac apex and lateral wall. Patients’ responses to AMI symptoms are different, of which hiccups can be characterized as a very rare one. The exact mechanism of hiccups is still unclear. Hiccup usually results from an irritation to the hiccup reflex arc. Acute vascular occlusion means corresponding myocardial necrosis. Probably materials released by necrotic myocardial cells stimulate branch of vagus nerve continually. Finally, hiccup happens and is difficult to terminate. In our case the situation gradually improved after achievement of TIMI1 flow in culprit vessel, strengthen of anticoagulant and stent implantation.

There are potential limitations of this case that need to be addressed. The first was how to access optimal anticoagulant intensity. He had a specific 2-year history of ischemic stroke with above-normal INR on admission. Aspirin plus clopidogrel can lead to significantly more major bleeding in ischemic stroke patients.^[11]^ INR variability is associated predictor of clinically relevant bleeding.^[12]^ Thus low molecular weight heparin was not prescribed on the base of dual antiplatelet drugs. The second consideration was whether we should maintain higher potassium concentration than average during acute phase of myocardial infarction. It was obvious that this patient had persistent gastrointestinal symptoms and poor appetite since admission. So electrolyte disorder was prone to happen. AMI accounts for ventricular arrhythmic events mainly. Below-average potassium concentration therefore predisposes to the occurrence of malignant arrhythmias.

Another feature is that some other medicines and remedies treating intractable hiccups need to be attempted in this case. Acupuncture has been used successfully in treating hiccup due to myocardial infarction.^[13]^ In this patient Zusanli acupoint massage was used but ineffectiveness. Possibly acupuncture treatment could be a preferable choice. Pharmacological therapy such as metoclopramide, which is reported to be effective at stopping intractable hiccups,^[14]^ could also be selected in this situation. Actually, metoclopramide had been tried temporarily in this patient for nausea-stopping whereas singultus did not improve. However, his thrombus in LAD may have been a large contribution factor to his particular symptom, which did not respond to any symptomatic treatment.

Overall, hiccup is only rarely described in the context of acute proximal LAD thrombosis. However, if this special symptom occurs intractably during disease progression, thrombus is revealed to be the probable cause, prompt opening of the criminal vessel should be performed besides strengthening of anticoagulant and antiplatelet.

## Author contributions

**Data curation:** Bo Zhang, Li Song, Suyu Yao.

**Formal analysis:** Hanxiang Gao.

**Funding acquisition:** Hanxiang Gao.

**Investigation:** Bo Zhang.

**Methodology:** Ming Bai.

**Project administration:** Zheng Zhang.

**Resources:** Li Song, Suyu Yao, Ming Bai.

**Writing – original draft:** Hanxiang Gao.

**Writing – review & editing:** Zheng Zhang, Ming Bai.
